# Genome-Wide Exploration and Expression Analysis of the CNGC Gene Family in Eggplant (*Solanum melongena* L.) under Cold Stress, with Functional Characterization of *SmCNGC1a*

**DOI:** 10.3390/ijms241713049

**Published:** 2023-08-22

**Authors:** Zheng Jiang, Lihui Du, Lei Shen, Jie He, Xin Xia, Longhao Zhang, Xu Yang

**Affiliations:** College of Horticulture and Landscape Architecture, Yangzhou University, Yangzhou 225009, China

**Keywords:** eggplant, gene family, cold stress, expression pattern, functional analysis

## Abstract

Eggplant (*Solanum melongena* L.) is an important economic crop, and to date, there has been no genome-wide identification and analysis of the cyclic nucleotide-gated channel (CNGC) gene family in eggplant. In this study, we identified the CNGC gene family in eggplant, and the results showed that 29 *SmCNGC* genes were classified into five groups, unevenly distributed across the 12 chromosomes of eggplant. The gene structure and motif analysis indicated that the *SmCNGC* family proteins may exhibit apparent preferences during evolution. Furthermore, our study revealed the presence of numerous light-responsive elements, hormone-responsive elements, and transcription factor binding sites in the promoter regions of *SmCNGC* genes, suggesting their significant role in environmental adaptability regulation. Finally, we analyzed the expression patterns of all *SmCNGC* genes under cold stress and found that *SmCNGC1a* was significantly upregulated under cold stress. Subcellular localization experiments indicated that this gene is located on the plasma membrane. Subsequently, its importance in the low-temperature response of eggplant was validated through virus-induced gene silencing (VIGS), and its protein interactome was predicted. In summary, our study provides a comprehensive understanding of the function and regulatory mechanisms of the CNGC gene family in eggplant, laying an important foundation for further research on cold adaptation in eggplant.

## 1. Introduction

Eggplant (*Solanum melongena* L.), a thermophilic vegetable belonging to the Solanaceae family, is extensively cultivated on a global scale. It is an important vegetable variety during the off season and is also one of the most popular and widely consumed vegetables [1]. Eggplant contains rich nutritional components, such as vitamins, minerals, and especially high levels of vitamin E, P, and iron. It also contains various bioactive compounds, offering health benefits and medicinal value [2]. Calcium ions (Ca^2+^) are indispensable for upholding the integrity of plant cell architecture [3]. They assume a pivotal role as both a critical cellular signal and a secondary messenger in numerous pathways, governing various responses to environmental stimuli, including plant hormones, temperature fluctuations, light exposure, and salt stress [4,5]. Cyclic nucleotide-gated channels (CNGCs) are non-selective cation channels situated on the cytoplasmic membrane. They play a crucial role in facilitating the transmembrane transport of monovalent cations, such as sodium and potassium, as well as divalent cations, like calcium and magnesium [6]. CNGCs in plants were first discovered in 1998 during the screening of a barley calcium-binding transporter (Hordeum vulgare CaM-binding transporter, HvCBT1), which revealed the existence of this non-selective cation channel [7]. CNGCs are widely distributed in animals and plants, serving as universal calcium ion channels in eukaryotes [8]. Prior research has substantiated the existence of CNGCs in both monocotyledonous and dicotyledonous plant species [9]. Preliminary investigations have suggested that CNGCs function as ligand-gated ion channels, facilitating the passage of calcium ions. These channels can be activated by cyclic nucleotides (cNMP) and their activity can be inhibited through binding with Ca^2+^/calmodulin (CaM) [10,11]. Structurally, CNGCs consist of six transmembrane domains (S1–S6), a pore region (P) located between the fifth and sixth domains, a C-terminal CaM-binding domain (CaMB), and a cyclic nucleotide-binding domain (CNBD) [12]. The CNBD, which represents a highly conserved region within CNGCs, is characterized by the presence of a cyclic nucleotide-binding cassette (PBC) and an adjacent unique “hinge” region. The PBC specifically interacts with the sugar and phosphate groups of the nucleotide ligand, while the adjacent “hinge” region contributes to the efficiency and selectivity of ligand binding [13]. Previous investigations have demonstrated that the carboxyl-terminal tails of specific plant CNGCs exhibit a Ca^2+^-dependent interaction with CaM [14,15,16]. In plants, CNGCs contain a conserved cNMP-binding domain that is considered a specific domain for identifying CNGCs [17]. Early studies proposed that plant CNGCs are regulated by cNMP gating. However, as research on calcium signaling has progressed, many scholars have questioned whether cNMP activation is necessary for CNGCs [18]. Recent studies have found that in *Xenopus laevis* oocyte cells, cAMP and cGMP can hyperpolarize activate calcium channels through heterologous assembly, without requiring an increase in cNMP levels [19]. Therefore, cNMP may only act as an auxiliary factor for CNGC subunits or exert its effects by modulating the membrane voltage or other regulators [19]. Furthermore, with further research, an increasing number of experimental findings indicate that the activation and regulation of CNGC channels rely more on phosphorylation modifications and the binding of CaM. The mechanisms underlying cNMP regulation of CNGCs require further investigation [20].

CNGCs regulate the growth of root tips in plants. Recent studies have shown that *CNGC5*, *CNGC6*, *CNGC9*, and *CNGC14* in *Arabidopsis thaliana* are involved in regulating the growth of root hairs [21]. *CNGC14* plays a role in maintaining cell integrity during polar growth of root hairs, and its mutation leads to abnormal growth of root hairs, including swelling, branching, and bursting at the tip [22]. Additionally, *CNGC5*, *CNGC6*, *CNGC9*, and *CNGC14* are also involved in maintaining the stability of unidirectional cell proliferation and cytoplasmic Ca^2+^ oscillations. In *CNGC14* mutants, the stability of cytoplasmic Ca^2+^ oscillations are severely impaired, and *cngc14/cngc6* and *cngc14/cngc9* double mutants lose the typical 30 s Ca^2+^ oscillation cycle [22]. In the double mutants of *cngc6* and *cngc9*, as well as in the single mutants of *cngc9*, this oscillation cycle still exists but with reduced stability [23]. These findings indicate that *CNGC14* plays a crucial role in these processes. Another study found that *CNGC5*, *CNGC6*, and *CNGC9* are essential Ca^2+^ channels in the structural growth of root hairs and auxin signal transduction in Arabidopsis. Mutations in these three genes lead to root hair growth defects, including shorter and defective root hairs. However, expressing any one of the CNGC subtypes individually or providing a high concentration of exogenous Ca^2+^ can restore this growth defect, whereas the supply of exogenous K^+^ cannot [24]. These three genes also exhibit Ca^2+^ permeation channel function in HEK293T cells. Cytosolic Ca^2+^ imaging and patch clamp data in root hairs indicate a significant reduction in the Ca^2+^ gradient and oscillation at the tip when *CNGC5*, *CNGC6*, and *CNGC9* are absent [24].

CNGCs play important roles in the growth and development of plant pollen tubes. The CNGC family in *Arabidopsis* comprises a total of 20 members [25]. Studies have shown that *AtCNGC16* contributes to the development of pollen grains in *Arabidopsis* under high-temperature conditions [26]. *AtCNGC7* and *AtCNGC8* share high amino acid sequence homology, and both have overlapping functions in the germination and development of pollen as the *cngc7/cngc8* double mutation leads to pollen sterility, confirming their roles in plant pollen development [27]. Further research has revealed that *CNGC7* or *CNGC8* interacts with *CNGC18* to form an inactive heterotetramer. However, when the Ca^2+^ concentration reaches its peak level, CaM dissociates from the *CNGC18-CNGC8* heterotetramer, relieving the inhibition of *CNGC18* by *CNGC8*. This pathway, regulated by Ca^2+^-CaM interaction with CNGC channels, facilitates autoregulatory feedback in calcium oscillations while facilitating pollen tube growth and plays an important role in fine-tuning the growth of pollen tubes [28]. Moreover, *CNGC18* exhibits asymmetric distribution on the plasma membrane at the growing tip of pollen tubes and possesses Ca^2+^ channel activity, which is crucial for pollen tube tip growth and guidance [29,30].

In recent years, with the increasing demand for year-round vegetables, particularly the rising requirements for eggplant production during the winter and spring seasons, eggplants frequently encounter cold stress during protected cultivation in winter and early spring, leading to cold damage, blossom and fruit drop, as well as poor fruit coloration. These phenomena severely affect the yield and quality of eggplants. Additionally, low-temperature stress hampers the growth and development of eggplants, resulting in stunted growth, yellowing and withering of leaves, and even plant death [31]. These occurrences not only directly impact the yield and quality of eggplants, but also cause economic losses and agricultural production instability for growers. Cold damage during the winter and spring seasons has become a bottleneck factor restraining the development of the eggplant industry, thereby imposing higher demands on the cold tolerance of eggplant cultivars during these seasons. Therefore, conducting research on the cold-tolerance mechanism of eggplants, exploring cultivation techniques to enhance their cold tolerance, and breeding new cold-tolerant eggplant varieties hold significant importance. In this study, we analyzed the genomic data of the eggplant CNGC gene family, identified 29 members of the *SmCNGCs* gene family, and conducted physicochemical property analysis, promoter analysis, phylogenetic tree construction, gene structure analysis, motif analysis, chromosome localization analysis, and collinearity analysis. We also investigated the expression patterns of *SmCNGCs* under different cold-stress conditions at different time points. Expression pattern analysis and subcellular localization were performed for the cold-stress-responsive gene *SmCNGC1a* in different tissues. Furthermore, the functionality of *SmCNGC1a* was validated using VIGS (virus-induced gene silencing) technology, and protein interaction networks were analyzed to explore the role of this gene under low-temperature stress in eggplants.

## 2. Results

### 2.1. Identification of the CNGC Genes in Eggplant

Based on the HMM file of the CNGC gene family, a search was conducted on the reference genome of eggplant. After manual screening and removal of redundant genes, a total of 29 *SmCNGC* genes were identified. Following the nomenclature guidelines for CNGC genes in *Arabidopsis* and referring to the information in the eggplant database, the identified 29 eggplant CNGC genes were named as *SmCNGC1a*-*SmCNGC24* (Table 1). The predicted lengths of *SmCNGC* proteins ranged from 482 amino acids (*SmCNGC9*) to 1074 amino acids (*SmCNGC7*). The majority of *SmCNGC* proteins had lengths concentrated in the range of 600–800 amino acids, with molecular weights varying from 55.40 kDa (*SmCNGC9*) to 122.28 kDa (*SmCNGC7*). The significant differences in amino acid length and molecular weight suggest potential structural variations among members of the *SmCNGC* gene family. Among the 29 *SmCNGC* proteins, seven had a theoretical pI value below 7, indicating a potential negative charge within the alkaline pH range. The remaining 22 *SmCNGC* proteins had theoretical pI values above 7, suggesting a positive charge within the acidic pH range. Additionally, 8 *SmCNGC* proteins had an instability index value below 40, indicating relative stability, while the remaining 21 proteins were relatively unstable. The predicted aliphatic index for *SmCNGC* proteins ranged from 88.78 (*SmCNGC18*) to 99.63 (*SmCNGC22*). The grand average of hydropathicity predicted that, with the exception of *SmCNGC13*, the remaining proteins were hydrophilic. The prediction of subcellular localization results indicated that the majority of *SmCNGC* proteins were located in the cell membrane (plasma membrane), consistent with their primary function as channel proteins. However, there were some special cases: *SmCNGC22* was found in the nucleus, *SmCNGC10* and *SmCNGC9* were located in the cytoplasm, and *SmCNGC23* was present in the chloroplast, suggesting potential diverse biological functions for these proteins.

### 2.2. Phylogenetic Analysis of SmCNGC Proteins

**Figure 1:**
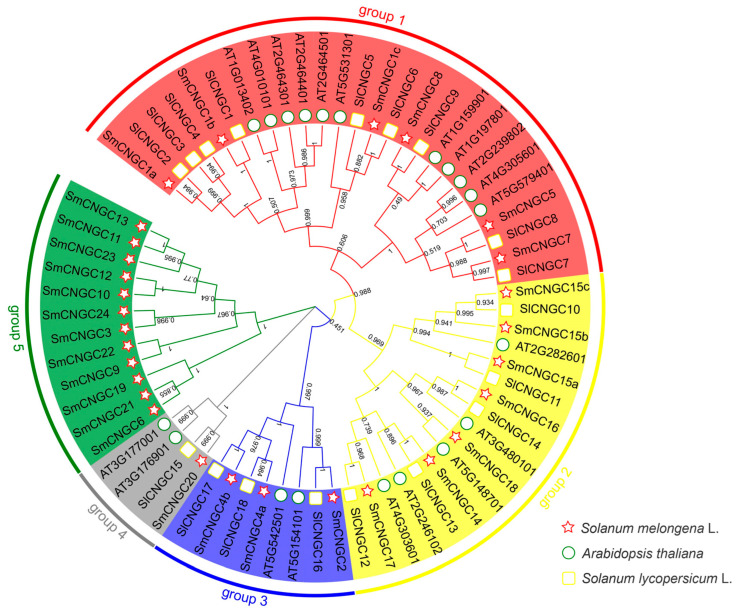
Phylogenetic relationships among the SmCNGCs, AtCNGCs, and SlCNGCs. The amino acid sequences of CNGC gene families in Arabidopsis, tomato, and eggplant were aligned using MEGA v7.0. After removing non-conserved gaps, the aligned sequences were used to construct a phylogenetic tree using the maximum likelihood (ML) method. Bootstrap values were set to 1000. Sm: eggplant; At: Arabidopsis; Sl: tomato. Different colors represent different groups.

To investigate the evolutionary relationships of the *SmCNGC* gene family, we constructed a phylogenetic tree of the CNGC protein family, including eggplant, Arabidopsis, and tomato (*Solanum lycopersicum* L.). The maximum likelihood (ML) method was employed to generate the phylogenetic tree with five groups (Figure 1). Group 1 consists of 6 eggplant CNGC proteins, 11 Arabidopsis CNGC proteins, and 9 tomato CNGC proteins. Group 2 comprises seven eggplant CNGC proteins, five Arabidopsis CNGC proteins, and five tomato CNGC proteins. Group 3 contains three eggplant CNGC proteins, two Arabidopsis CNGC proteins, and three tomato CNGC proteins. Group 4 consists of one eggplant CNGC protein, two Arabidopsis CNGC proteins, and one tomato CNGC protein. Group 5 exclusively includes 12 eggplant CNGC proteins, indicating a relatively distant evolutionary relationship with Arabidopsis and tomato. Based on the evolutionary analysis results, it can be inferred that CNGC maintains a high degree of stability throughout the process of species evolution, suggesting its conserved and indispensable role in organisms.

### 2.3. SmCNGC Gene Structures and the Conserved Motifs Analyses

**Figure 2:**
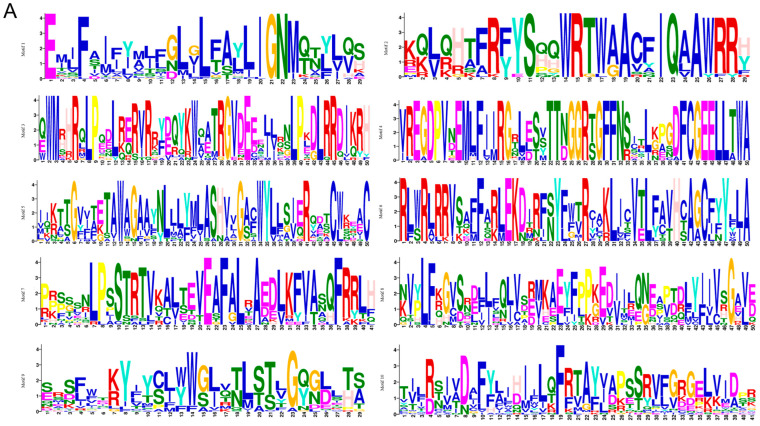

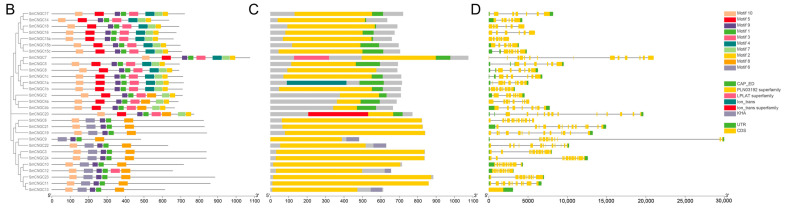
*SmCNGC* gene structures and the conserved motifs analyses. (**A**) Amino acid composition of each motif. (**B**) Motif compositions. (**C**) Conserved domains. (**D**) Gene structure. The motif logos were generated using the MEME Suite web server, while the remaining figures were generated using Tbtools.

Motif analysis was conducted on the CNGC gene family in eggplant. The results revealed that the encoded proteins of this family possess 10 conserved motifs (motif 1–10, Figure 2A). Members within the same subfamily exhibited similar distribution patterns of these conserved motifs. Motif 10 was identified as a shared motif among 28 *SmCNGC* members, while motif 1 was absent in *SmCNGC9*. Furthermore, it was observed that CNGC members in group 5 displayed unique regularity in the motifs they contained, with motif 6 being exclusive to this group (Figure 2B). Structural domain analysis indicated that most of the genes contained the PLN03192 superfamily domain. However, *SmCNGC1a* lacked this domain, which suggests that there might be functional differences associated with this gene (Figure 2C). Gene structure analysis, as shown in the diagram on the right, revealed that the number of coding sequences (CDSs) and untranslated regions (UTRs) were similar within the same subfamily. Additionally, *SmCNGC13* displayed significant structural differences compared to other members, indicating possible variations in gene structure during the process of evolution (Figure 2D). 

### 2.4. Chromosome Localization and Cis-Acting Elements Prediction of SmCNGCs

**Figure 3:**
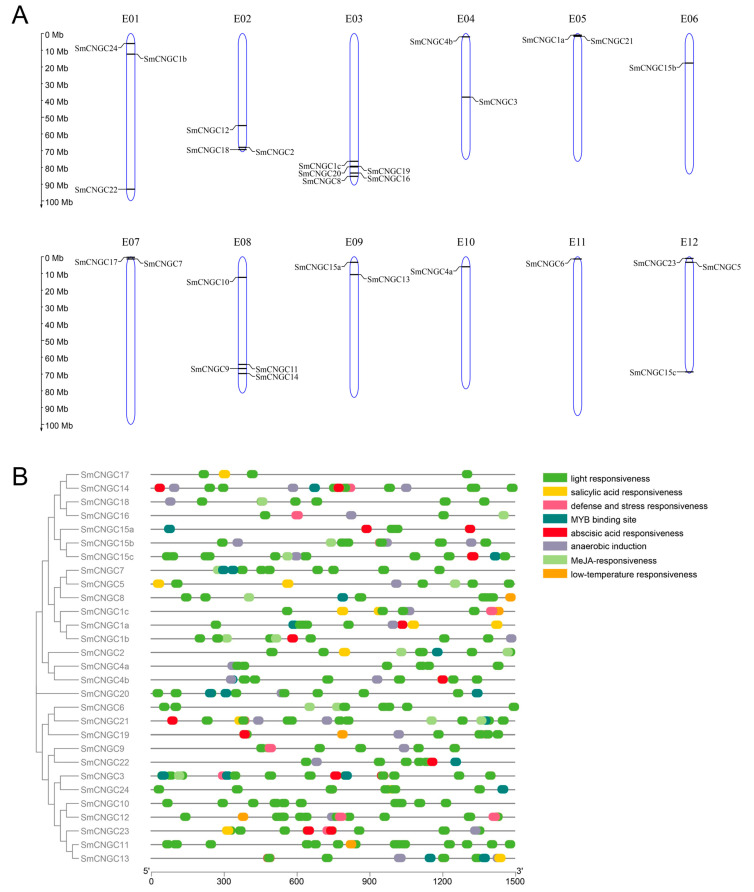
Analysis of Chromosomal Location and Cis-Regulatory Elements of the *SmCNGC* Gene. (**A**) Chromosomal localization of *SmCNGCs*. The blue bars represent the length of each chromosome, reflecting their relative sizes. The *SmCNGCs* are indicated on the chromosomes using their respective gene names. The placement of gene names along the chromosomes signifies their positions within the genome of eggplant. (**B**) Prediction of cis-regulatory elements in the upstream regions of *SmCNGC* genes. Different colors represent different cis-regulatory elements, with each color corresponding to a specific functional motif. The genes are arranged on the left side of the figure according to their evolutionary relationships. The color legend on the right side of the figure indicates the different cis-regulatory elements, with the colors arranged in descending order of their occurrence frequency in *SmCNGCs*.

Chromosomal localization analysis revealed that members of the *SmCNGC* gene family are distributed across all 12 chromosomes of eggplant. Most of these genes are located at the ends of the chromosomes. Chromosome 3 harbors the highest number of CNGC gene family members, with five genes. Chromosome 8 contains four CNGC gene family members. Chromosomes 1, 2, and 12 have three members each, while chromosomes 4, 5, 7, and 9 have two members each. Chromosomes 6, 10, and 11 have only one member of the CNGC gene family (Figure 3A). Promoter analysis predicted the presence of numerous cis-regulatory elements. The results indicate that the promoters of *SmCNGC* genes contain a substantial number of light-responsive elements, anaerobic-induction elements, hormone-responsive elements, and MYB transcription factor binding sites. All *SmCNGC* gene promoters contain light-responsive elements, suggesting their potential involvement in light signal regulation. Additionally, we found that a significant portion of *SmCNGC* genes contain methyl jasmonate, salicylic acid, or abscisic-acid-responsive elements, indicating their potential role in responding to these hormones and regulating plant growth and development. Nearly half of the *SmCNGC* genes contain MYB transcription factor binding sites, suggesting that SmCNGC may be directly regulated by MYB transcription factors, thus modulating ion channel activity and signal transduction (Figure 3B).

### 2.5. Collinearity Analysis of SmCNGCs

**Figure 4:**
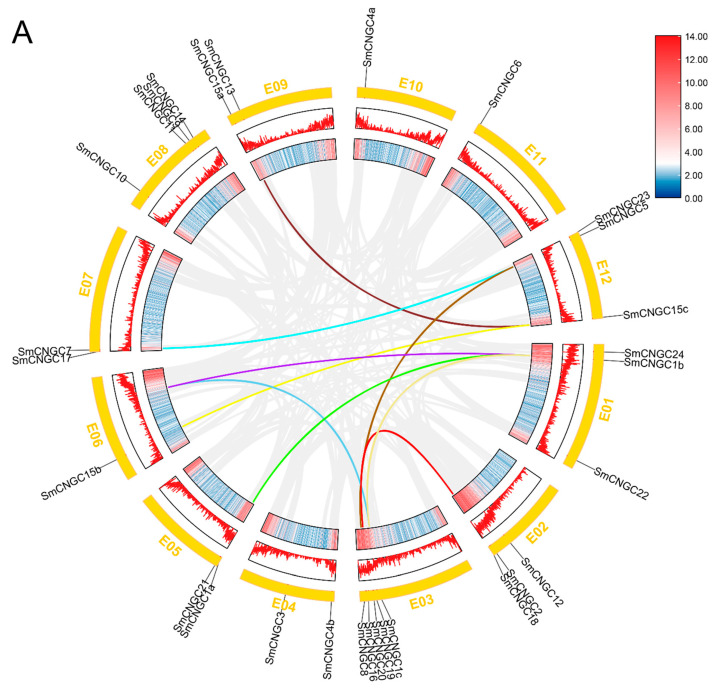

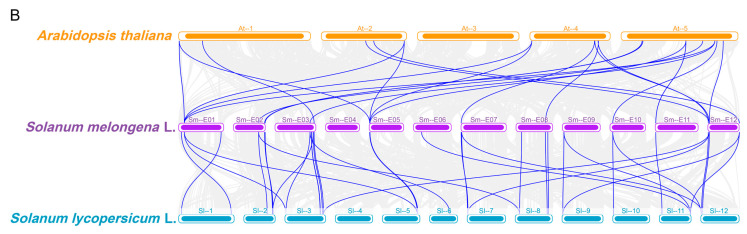
Collinearity analysis of *SmCNGCs*. (**A**) Chromosome locations and inter-chromosomal associations of *SmCNGC* genes. The colored lines represent different sets of collinear genes. The gene density in the chromosome is also depicted in the graph, with red indicating high density and blue indicating low density. (**B**) Collinearity analysis of CNGC genes between eggplant and the other two representative plants. The syntenic gene pairs were linked by blue lines.

To elucidate the origin and evolutionary relationship of the *SmCNGC* gene family, we analyzed the gene duplication events of *SmCNGC*. The results showed that the *SmCNGC* gene family has a total of nine pairs of gene sequence duplication events, namely *SmCNGC13/SmCNGC15c*, *SmCNGC7/SmCNGC23/SmCNGC17*, and *SmCNGC15b/SmCNGC15c*, etc. (Figure 4A). This indicates that the *SmCNGC* genes have undergone multiple duplication events during the evolutionary process, which facilitated the rapid expansion of the *SmCNGC* gene family. In addition, we performed collinearity analysis of the genomes of eggplant, Arabidopsis, and tomato using MCScanX v1.5.1. The results showed that there are 22 pairs of collinearity relationships between eggplant and Arabidopsis CNGC genes, and 34 pairs of collinearity relationships between eggplant and tomato. It is noteworthy that eggplant’s chromosome 3 (E03) has the highest number of collinear gene pairs (Figure 4B).

### 2.6. Expression Patterns of SmCNGC Genes in Cold Stress

**Figure 5:**
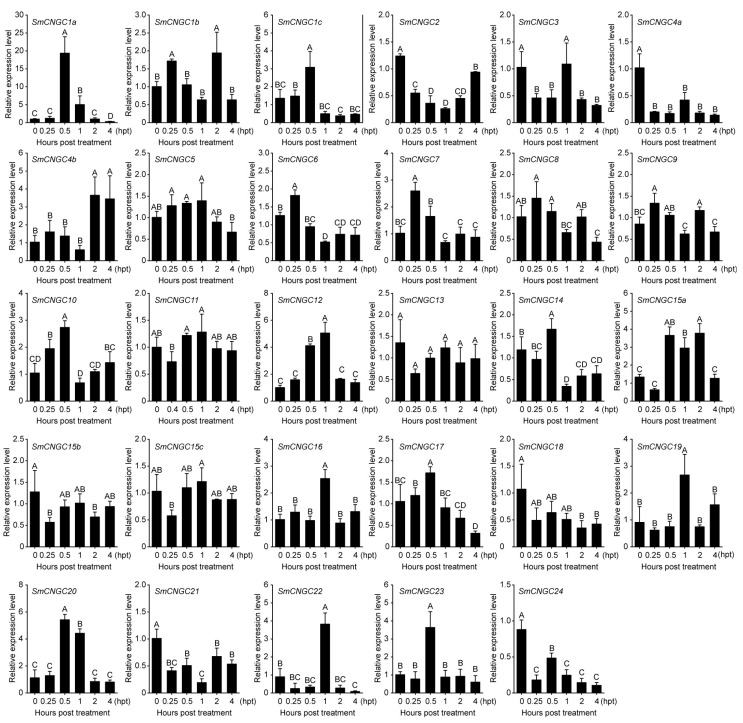
Expression patterns of *SmCNGC* genes under different time points of 4 °C cold stress. The bar graph represents the expression patterns of *SmCNGC* genes under different time points of cold stress. The y-axis represents the relative expression levels, and the x-axis represents the time points of cold stress. Data are means ± standard deviation from four biological replicates. Different capital letters between samples denote significant differences according to one-way ANOVA and Tukey’s test (*p* < 0.01). The error bars represent the standard deviation.

To investigate the expression patterns of the *SmCNGC* genes under cold stress in eggplant and evaluate their roles during cold stress, we employed qRT-PCR to detect the expression levels of *SmCNGC* genes at different time points (0, 0.25, 0.5, 1, 2, and 4 h) of cold stress. The results revealed that the *SmCNGC* genes exhibited distinct expression patterns in response to cold stress at different time intervals. Specifically, the expression levels of *SmCNGC1a*, *SmCNGC1c*, *SmCNGC7*, *SmCNGC12*, and *SmCNGC20* showed an initial increase followed by a decrease, suggesting their potential importance in the response to cold stress. This initial upregulation of expression might be associated with the rapid adaptation to environmental changes induced by cold stress and could be involved in the regulation of ion channels and signal transduction within the cells. As the duration of cold stress continued, the expression levels of these genes gradually decreased, which could indicate cellular acclimation to the cold-stress conditions or involvement of negative feedback regulatory mechanisms. Furthermore, the expression levels of *SmCNGC4a*, *SmCNGC6*, *SmCNGC18*, and *SmCNGC24* decreased with prolonged cold stress, indicating their negative regulatory roles in eggplant’s adaptation to cold stress. Interestingly, we observed a trend: initially decreased expression levels followed by increased expression levels for *SmCNGC2* and *SmCNGC21* (Figure 5). These experimental findings suggest that the *SmCNGC* genes may exhibit temporal specificity during the plant’s response to cold stress. Of note, we observed a significant upregulation of *SmCNGC1a* at 0.5 h of cold stress, indicating its potential crucial role in the response of young eggplant seedlings to cold stress. Subsequent experiments will be conducted to further investigate this gene.

### 2.7. Expression Patterns of SmCNGC1a under Various Stress Conditions

**Figure 6:**
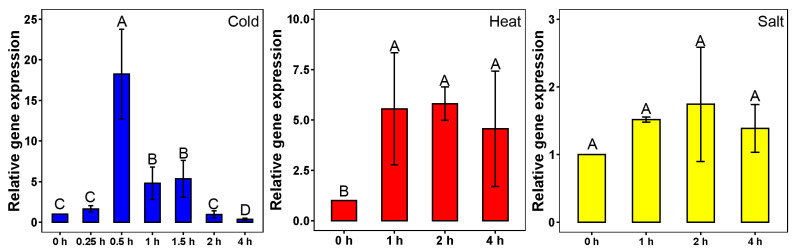
Expression patterns of SmCNGC1a under different stresses. Gene expression was analyzed using qPCR, with 0 h as the control. The blue columns represent cold stress, the red columns represent heat stress, and the yellow columns represent salt stress. Data are means ± standard deviation from four biological replicates. Different capital letters between samples denote significant differences according to one-way ANOVA and Tukey’s test (*p* < 0.01). The error bars represent the standard deviation.

To comprehensively investigate the expression patterns of *SmCNGC1a* under various stress conditions, we conducted analyses of its response to cold stress, heat stress, and salt stress. The qPCR results revealed distinct expression profiles of *SmCNGC1a* under these diverse stress scenarios. Under cold stress, the transcription level of *SmCNGC1a* rapidly increased during the initial phase of exposure to cold stress, reaching a significant level after 0.5 h, followed by a gradual decline during sustained cold stress. This transient nature implies that *SmCNGC1a* may be involved in the early perception and response phases of cold stress. Under heat stress, the transcription level of *SmCNGC1a* showed a sustained upregulation, maintaining a significant elevation compared to the untreated control. This sustained upregulation suggests that *SmCNGC1a* could potentially play a role in mediating eggplant’s response to heat stress. In contrast to cold and heat stress, the response of *SmCNGC1a* to salt stress exhibited a different expression pattern. Although there was a slight increase in gene expression, it was not statistically significant. This indicates a potential subtle role of *SmCNGC1a* in the regulation of salt-stress response (Figure 6).

### 2.8. Subcellular Localization and Tissue-Specific Expression Patterns of SmCNGC1a

**Figure 7:**
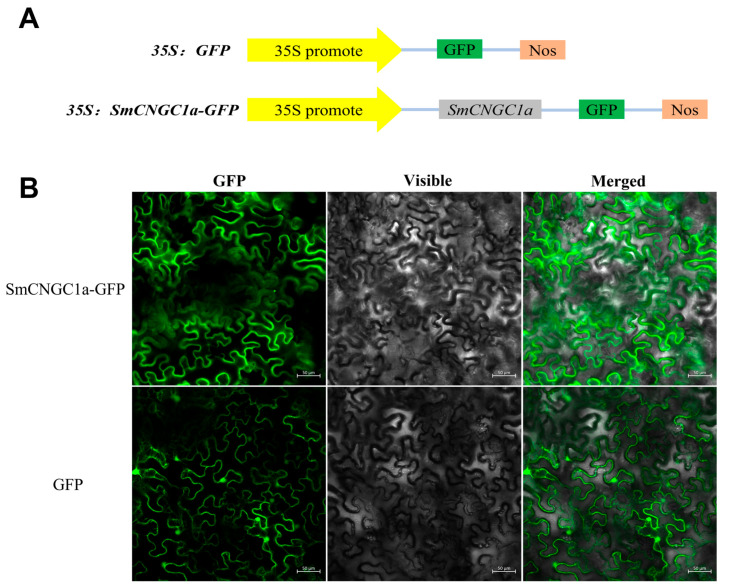
Subcellular localization of *SmCNGC1a*. (**A**) Construction of the *SmCNGC1a* vector. (**B**) 35S:*SmCNGC1a*-GFP and 35S:GFP (control) were transformed into *N. benthamiana* epidermal cells using *Agrobacterium*-mediated transformation and injected. Results were observed using confocal microscopy 48 h after transformation. Scale bars = 50 μm.

**Figure 8:**
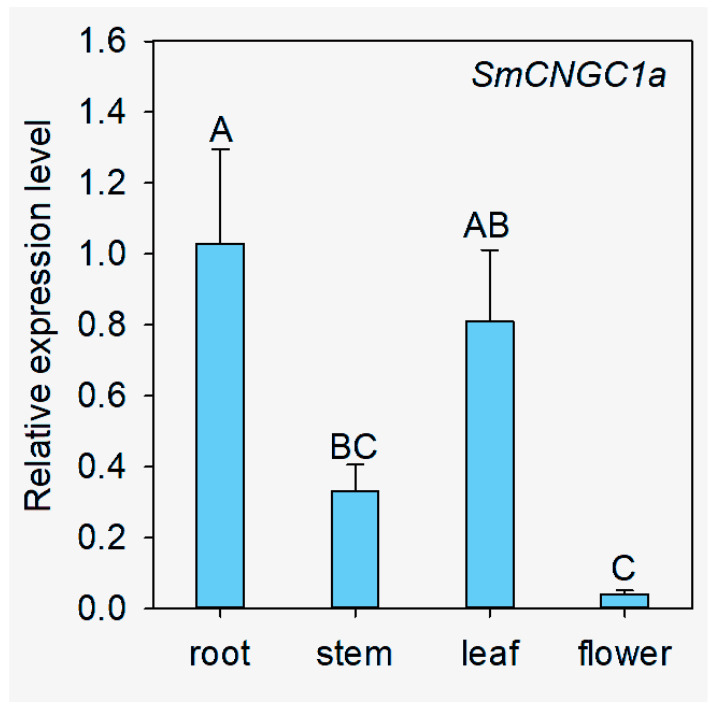
Expression levels of *SmCNGC1a* in different tissues of eggplant. Data are means ± standard deviation from four biological replicates. Different capital letters between samples denote significant differences according to one-way ANOVA and Tukey’s test (*p* < 0.01), The error bars represent the standard deviation.

To investigate the subcellular localization and expression patterns of *SmCNGC1a* in different plant tissues, we cloned *SmCNGC1a* into the pCambia1300-35S-EGFP plant expression vector containing the 35S promoter and GFP reporter gene (Figure 7A). The recombinant constructs, 35S:*SmCNGC1a*-GFP and 35S:GFP (control), were transformed into *N. benthamiana* epidermal cells using *Agrobacterium*-mediated transformation and injected. After 48 h, the fluorescence signals of the genes in the leaves were observed using a laser scanning confocal microscope. The results indicate that the fluorescence signal of *SmCNGC1a*-GFP was localized to the plasma membrane of the cells, while the control group (GFP) exhibited a fluorescence signal distributed throughout the entire cell, suggesting that *SmCNGC1a* is localized to the plasma membrane (Figure 7B). Furthermore, we performed qRT-PCR to detect the expression patterns of *SmCNGC1a* in different tissues of eggplant (root, stem, leaf, and flower). The results revealed that *SmCNGC1a* exhibited relatively higher expression levels in the root and leaf, and relatively lower expression levels in the stem and flower, suggesting its possible association with environmental adaptation in roots and leaves (Figure 8).

### 2.9. Silencing of SmCNGC1a Reduced Eggplant Tolerance to Cold Stress

**Figure 9:**
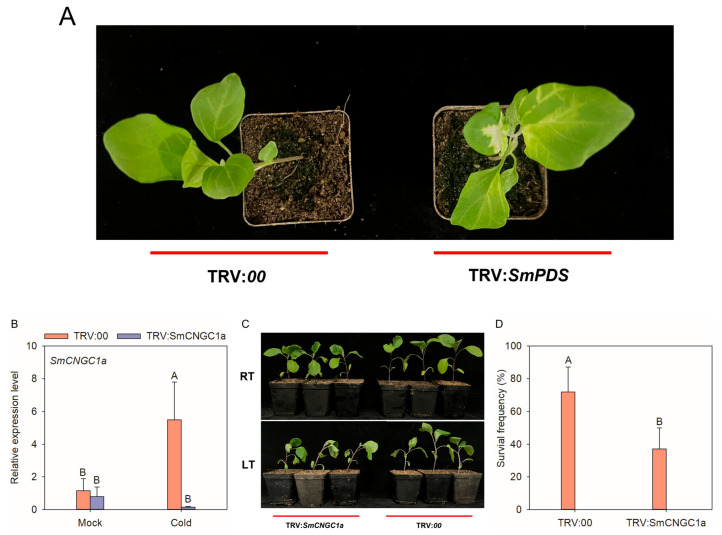

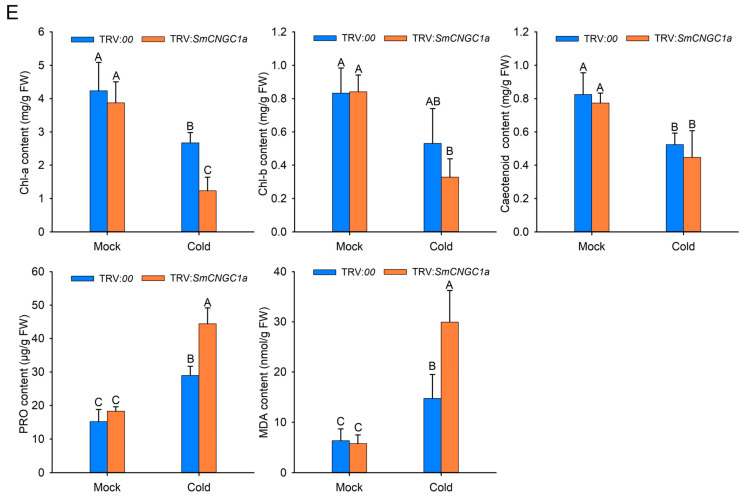
Effects of *SmCNGC1a* silencing on eggplant responses to cold stress (**A**) TRV: the albino phenotype of *SmPDS* indicates the success of the silencing system. (**B**) Silencing efficiency of *SmCNGC1a* in plants under cold stress based on a qRT-PCR assay. (**C**) Phenotype of *SmCNGC1a*-silenced and control plants challenged with cold stress at 2 h post-treatment. RT: 25 °C; LT: 4 °C. (**D**) Survival frequencies of *SmCNGC1a*-silenced and control plants subjected to cold stress at 2 h post-treatment. (**E**) Contents of chlorophyll a, chlorophyll b, carotenoids, proline, and malondialdehyde in *SmCNGC1a*-silenced and control plants after 2 h of cold stress. Chl-a: chlorophyll a; Chl-b: chlorophyll b; PRO: proline; MDA: malondialdehyde; FW: fresh weight. Different capital letters between samples denote significant differences according to one-way ANOVA and Tukey’s test (*p* < 0.01).

To further investigate the function of *SmCNGC1a* in the cold-stress response of eggplant, we employed the VIGS method to silence *SmCNGC1a*. *Agrobacterium* infiltration solution was injected into the leaves of “JS221” seedlings, and 21 days later, the JS221 leaves injected with TRV:*SmPDS* exhibited pronounced chlorosis symptoms (Figure 9A), while no significant phenotype was observed in the control plants. Subsequently, we used qRT-PCR to examine the expression levels of the *SmCNGC1a* gene in the roots of silenced and control plants under cold stress to calculate the silencing efficiency. The results showed that the relative expression level of *SmCNGC1a* was significantly lower in the silenced plants compared to the control plants. In the control group, the expression of *SmCNGC1a* in the leaves was significantly upregulated under cold stress, while the expression level of *SmCNGC1a* in the silenced plants was suppressed (Figure 9B). These findings indicate successful silencing of *SmCNGC1a*. Furthermore, after 2 h of cold stress, the silenced plants exhibited more severe leaf wilting, stem bending, and loss of turgidity (Figure 9C), and the survival rate of the plants also significantly decreased (Figure 9D), suggesting that the silencing of *SmCNGC1a* reduced the cold tolerance of JS221, thus preliminarily confirming its positive regulatory role in the cold-stress response of eggplant. We also monitored the contents of chlorophyll a, chlorophyll b, carotenoids, proline, and malondialdehyde in the leaves of plants before and after silencing. The results revealed a significant decrease in the levels of photosynthetic pigments (chlorophyll a and chlorophyll b) compared to the control, accompanied by a substantial increase in proline and malondialdehyde content (Figure 9E). This suggests that the silencing of SmCNGC1a significantly affects the accumulation of proline and malondialdehyde in eggplant leaves, leading to a more pronounced degradation of chlorophyll a and chlorophyll b when compared to non-silenced plants.

### 2.10. Analysis Interaction Network of SmCNGC1a in Eggplant

**Figure 10:**
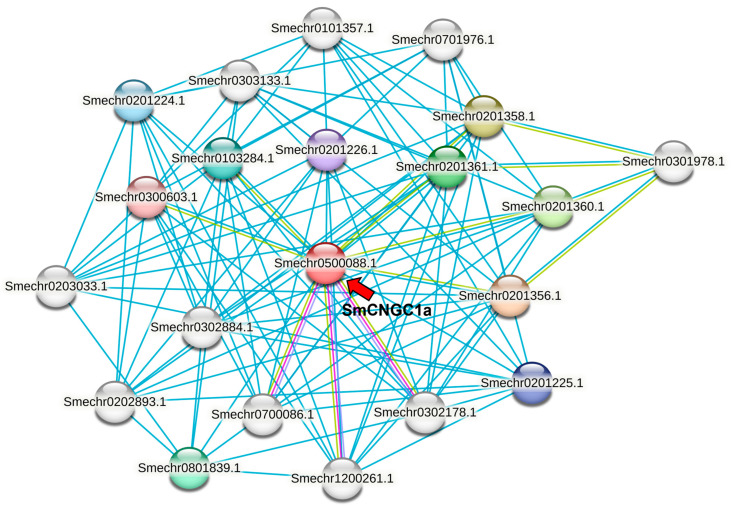
Interaction network analysis of *SmCNGC1a*. Nodes represent different proteins, with colored nodes indicating proteins that may have first shell of interactions with *SmCNGC1a*, and uncolored nodes indicating proteins that may have second shell of interactions with *SmCNGC1a*. Lines represent known or predicted protein–protein interactions, with blue lines indicating interactions predicted from homologous genes in other species, red lines indicating interactions that have been experimentally validated, green lines indicating interactions predicted using text mining methods, and purple lines indicating the presence of homology between the connected proteins. The “Smechr0500088.1” referred to by the red arrow is identified as SmCNGC1a.

To further investigate the function of *SmCNGC1a*, we utilized STEING v11.5 to predict and analyze its interaction network in eggplant. The results revealed that *SmCNGC1a* interacts with proteins related to defense response by callose deposition in the cell wall, regulation of anion channel activity, and receptor-mediated endocytosis (Smechr0201361.1, Smechr0201358.1, Smechr0201356.1, Smechr0201360.1). Additionally, it interacts with proteins associated with protein serine/threonine kinase activity, adenyl ribonucleotide binding, and ATP binding (Smechr0201224.1, Smechr0801839.1, Smechr0201360.1, Smechr0103284.1, Smechr0201226.1, Smechr0201225.1). The predicted interacting proteins’ KEGG pathways indicate their potential involvement in the plant MAPK signaling pathway and plant–pathogen interaction. The results of STEING local network cluster analysis suggest that these proteins may participate in cGMP-binding defense response by cell wall thickening and plant MAPK signaling pathway (Figure 10).

## 3. Discussion

With the increasing exploration of reference genomes in various plant species, many gene families have been identified; however, the CNGC gene family in eggplant remains unknown. In this study, we identified the CNGC gene family in eggplant and conducted comprehensive bioinformatics analyses of its physicochemical properties, phylogenetic evolutionary relationships, chromosome localization, gene structure, cis-acting elements, and gene duplication events. Subsequently, we analyzed the expression patterns of the CNGC gene family under cold stress and identified a significantly upregulated gene, *SmCNGC1a*, in response to cold stress. We further analyzed its subcellular localization and tissue-specific expression patterns. Finally, we employed VIGS technology to validate the function of this gene and analyzed its protein interaction network. Our physicochemical property analysis revealed that most members of the *SmCNGC* family are hydrophilic proteins localized in the plasma membrane, which confirms their role as ion channel proteins. Phylogenetic evolutionary analysis showed that the *SmCNGC* family has relatively more members compared to Arabidopsis and tomato, with a distinct branch exhibiting a distant evolutionary relationship with other species. This indicates that CNGC proteins in eggplant may have undergone unique evolutionary processes, possibly due to differences in the environmental conditions for eggplant survival during natural evolution compared to Arabidopsis and tomato, leading to adaptive evolution of the genes. Motif analysis identified a total of 10 motifs, among which the majority of *SmCNGC* proteins exhibit 8 conserved motifs characterized by similar sequences. However, a unique motif 6 is present in another branch, while motif 5 is absent, suggesting that this subset of *SmCNGC* proteins may have distinct preferences and potentially exert specialized functions during evolution.

According to reports, Ca^2+^ channels play a crucial role in plant responses to temperature stress [32]. Studies conducted on rice (*Oryza sativa* L.), cabbage (*Brassica oleracea* L.), tobacco (*Nicotiana benthamiana*), and mango (*Mangifera indica* L.) have shown that the expression of CNGC gene family undergoes changes under cold-stress conditions. For instance, 10 *OsCNGCs* in rice [33], 13 *BoCNGCs* in cabbage [34], 10 *NtabcCNGCs* in tobacco [35], and the genes *MiCNGC15* and *MiCNGC15II* in mango fruit peel [36] were found to be upregulated under cold stress. Additionally, a study in 2020 identified 15 *ZjCNGCs* in the jujube (*Ziziphus zizyphus*) genome, where *ZjCNGC2*, *8*, *10*, and *ZjCNGC15* showed downregulation within 24 h of cold-stress treatment, while the expression levels of *ZjCNGC4* and *ZjCNGC12* increased approximately four-fold and two-fold, respectively, after 1 h of cold treatment [37]. According to the research on temperature-stress treatment in Chinese cabbage (*Brassica rapa pekinensis*), it was found that the expression of *BrCNGC1*, *2*, *3*, *10*, *17, 22*, *23*, *27*, and *BrCNGC29* was upregulated under low-temperature stress [38]. Apart from cold stress, CNGCs have also been found to be involved in regulating heat stress. Specifically, the genes *AtCNGC6* and *AtCNGC2* are involved in plant response to heat stress and are closely associated with plant thermotolerance. Under high-temperature induction, *AtCNGC6* triggers Ca^2+^ influx and the adaptive expression of heat-shock proteins. Mutants of *AtCNGC2*, namely *cngc2-1* and *cngc2-2*, exhibit enhanced tolerance to heat stress and accumulate more heat-responsive proteins [39,40]. Conversely, interference with *CNGCb* (a homologous gene of *AtCNGC2*) from the moss Physcomitrella patens leads to a super-thermosensitive phenotype in plants [41]. In rice, *OsCNGC14* and *OsCNGC16* also play important roles in plant thermotolerance. Mutants *cngc14* and *cngc16* show lower survival rates under high and low-temperature stress, and the extent of heat-stress induction and inhibition of certain genes is altered in the cngc16 mutant. Furthermore, the absence of *OsCNGC14* or *OsCNGC16* reduces or eliminates the cytosolic Ca^2+^ signals induced by temperature stress [42]. In this study, we found numerous light-responsive elements, hormone-responsive elements, and transcription factor binding sites in the promoter region of the *SmCNGC* gene, indicating that this gene family may respond to environmental signals and stress, potentially playing an important role in regulating eggplant’s environmental adaptation. The expression pattern analysis of *SmCNGC* genes under cold stress also suggests that many genes in this family can respond to low-temperature signals. Specifically, we observed significant upregulation of *SmCNGC1a* under cold stress, and this finding was further validated through VIGS experiments, highlighting its importance in eggplant’s response to low temperatures.

In addition, we predict complex interactions between *SmCNGC1a* and various proteins in eggplant, including cell wall defense response proteins, anion channel activity regulatory proteins, and serine/threonine-kinase-related proteins. Cell wall defense response proteins participate in the synthesis and deposition of pectin, enhancing the defensive capacity of plant cell walls. They are important mechanisms for plants to resist stress and adversity [43]. Anion channel activity regulatory proteins, similar to CNGCs, constitute a class of membrane protein channels that can regulate intracellular ion balance and signal transduction [44]. During stress and adversity, these proteins may interact synergistically with CNGCs to resist stress. Serine/threonine kinases are a class of enzyme proteins that phosphorylate serine (Ser) or threonine (Thr) residues in target proteins and participate in the regulation of various signal transduction pathways [45]. We speculate that *SmCNGC1a* may interact with the aforementioned proteins to transmit cold-stress signals and activate cold-stress-defense-related genes. However, this speculation requires further experimental validation. It is worth noting that although our study confirms the positive regulatory role of *SmCNGC1a* in cold tolerance in eggplant, there are still many unresolved questions that require further investigation. For example, a deeper understanding of the process by which *SmCNGC1a* transmits cold-stress signals and clarification of its regulatory relationships with other genes, as well as the detailed mechanisms of the regulatory pathways, are needed. Our study provides a reference for further research on cold-tolerance genes in eggplant.

## 4. Materials and Methods

### 4.1. Identification of CNGC Gene Family in Eggplant Genome

The reference genome, coding sequences (CDS), and protein sequences of eggplant were obtained from the Eggplant Genome Database (Table 2). Hidden Markov model (HMM) profiles for the cyclic nucleotide-binding domain (cNMP, PF00027) and the ion transport protein domain (iTP, PF00520) used by the eggplant CNGC gene family were obtained from the InterPro protein family database (Table 2). The HMM profiles were searched using HMMER v3.0 software to identify genes containing both cNMP and iTP domains. These genes were then extracted, and further manual verification was performed using the NCBI CDD (Table 2) to ensure the inclusion of genes with both conserved domains and to remove pseudogenes. The final set of *SmCNGC* gene family members was obtained. The molecular weight, theoretical isoelectric point, instability index, aliphatic index, and average hydrophilicity of all the genes in the family were predicted using the Expasy ProtParam tool (Table 2). Additionally, the subcellular localization of the *SmCNGCs* was predicted using the WoLF PSORT subcellular localization prediction tool (Table 2).

### 4.2. Multiple Sequence Alignment and Phylogenetic Analysis

The protein sequence information of the Arabidopsis CNGC gene family was obtained from TAIR (Table 2). The protein sequences of the tomato CNGC gene family were referenced based on the identification results from previous studies conducted by other researchers [17] and obtained from the tomato genome database (Table 2). The amino acid sequences of CNGC gene families in Arabidopsis, tomato, and eggplant were aligned using MEGA v7.0. After removing non-conserved gaps, the alignment results were used to construct a phylogenetic tree using the maximum likelihood (ML) method with a bootstrap value set at 1000. The generated phylogenetic tree file was imported into Evolview v3 (Table 2) for visualization.

### 4.3. Gene Structures and Conserved Motifs Analysis

The genomic annotation file (.gff) for eggplant was obtained from the Eggplant Genome Database. The software TBtools v1.120 was used to extract gene length and positional information from the genomic annotation file of *SmCNGCs*. The extracted data were then visualized. The amino acid sequence motifs of *SmCNGCs* were analyzed using MEME (Table 2), with the search model set as “anr” and a minimum motif length of 6 and a maximum motif length of 50. Only the top 10 most reliable pieces of motif information were retained. The analysis results were visualized using MEME and Tbtools software [46].

### 4.4. Gene Distribution and Cis-Acting Elements Prediction

After extracting the chromosome position information of *SmCNGCs* using TBtools, the extracted results were imported into MapGene2Chromosome v2.1 (Table 2) to generate a chromosome location map of the gene family members. The promoter sequences of the *SmCNGCs* family were obtained using TBtools, specifically the 1500bp upstream of the gene’s start codon. These promoter sequences were then analyzed using the PlantCARE website (Table 2) to identify and retain regulatory elements associated with environmental response, hormone response, and transcription factor binding sites that occur frequently. The retained elements were visualized using TBtools [46].

### 4.5. Collinearity Analysis

To uncover intraspecies microsynteny groups in eggplant, we utilized the MCScanX plugin integrated within the TBtools software to perform collinearity analysis. Subsequently, the Circos plugin in TBtools was employed to generate circos plots depicting the microsynteny groups. Additionally, we employed MCScanX to analyze the co-occurrence relationships between the CNGC gene family in eggplant and those in Arabidopsis thaliana and tomato. Visualization was carried out using TBtools [46].

### 4.6. Plant Materials and Treatments

The plant materials used in this experiment were eggplant variety “JS221” and *N. benthamiana*. Seeds were germinated by placing them on moist filter paper in Petri dishes, followed by cultivation in a growth chamber. The growth chamber was kept at a constant temperature of 25 °C, with a relative humidity of 60–70% and a photoperiod of 16 h of light (800 µmol m^−2^ s^−1^) followed by 8 h of darkness. After germination, the seedlings were transplanted into seedling trays and subsequently transferred to small pots once the cotyledons had expanded, allowing for further growth. The plants were cultivated at a temperature of 25 °C and a photoperiod of 16 h of light (800 µmol m^−2^ s^−1^) followed by 8 h of darkness until they reached the 4–6 leaf stage for subsequent experiments. Cold-stress treatment was conducted in the growth chamber at a temperature of 4 °C and heat-stress treatment was conducted in the growth chamber at 42 °C, while the other conditions remained unchanged. After the treatment, the eggplant leaves were washed with ddH_2_O. Salt-stress treatment was conducted following the methods described in previous studies, with sampling focused on the root [47]. The samples were cryogenically preserved by immersing them in liquid nitrogen and then stored at a temperature of −80 °C to facilitate further analysis.

### 4.7. Subcellular Localization

*SmCNGC1a* was constructed into the pCambia1300-35S-EGFP plant overexpression vector to generate a fusion vector containing 35S: *SmCNGC1a*-GFP, which was then transformed into *Agrobacterium* strain *GV3101*. The composition of *Agrobacterium* suspension culture medium refers to previous studies in the field [47]. Healthy *N. benthamiana* were selected as the experimental material. Using a sterile syringe, the leaf surface of *N. benthamiana* was injected with OD_600_ = 0.8 suspension of pCambia1300-35S-EGFP empty vector and pCambia1300-*SmCNGC1a*-GFP separately, avoiding the leaf veins. This process was repeated three times. Subsequently, the injected *N. benthamiana* were cultivated in a growth chamber under appropriate lighting conditions for 48 h. The *N. benthamiana* leaves were then cut into suitable sizes, rinsed with sterile water, and mounted on glass slides. The fluorescence signals of the genes in the leaves were observed using a laser scanning confocal microscope (LSM 880NLO; Leica Microsystems, Wetzlar, Germany) to determine the cellular localization of *SmCNGC1a*.

### 4.8. Functional Analysis of SmCNGC1a Based on VIGS Method

The VIGS method referred to in previous studies [47] was employed. RNA was isolated from the leaves of pTRV2-*SmCNGC1a*-silenced plants and pTRV2 negative control plants, followed by qRT-PCR analysis to assess the silencing efficiency of *SmCNGC1a*. Cold-stress treatment was conducted according to the procedure outlined in Section 2.6. After 2 h treatment, the phenotypes of eggplant seedlings were observed under different experimental conditions, and the survival rates were calculated.

### 4.9. Gene Expression Analysis by qPCR

To analyze the transcriptional expression levels of the target gene, qPCR was performed following the method established by previous studies [47]. Four biological replicates were used to detect the transcript expression levels of the target gene. The primers used in this study are listed in Table 3.

### 4.10. Physiological Parameter Determination

The determination method of photosynthetic pigments is as follows: Firstly, 0.2 g of leaf samples were weighed into a mortar, and 12 mL of 95% ethanol was added. The samples were ground until the tissue turned white and left to stand for 3–5 min. Then, the mixture was filtered into a 25 mL brown volumetric flask using filter paper. The filter paper and residue were rinsed several times until no residues remained. Finally, the volume was adjusted to a fixed volume with ethanol and shaken well. The absorbance values (A_665_, A_649_, and A_470_) of the chlorophyll extract were measured at wavelengths of 665 nm, 649 nm, and 470 nm, respectively. The concentrations (mg/L) of chlorophyll a, chlorophyll b, and carotenoids were calculated using the following formulas: Ca = 13.95 × A6656.88 × A649; Cb = 24.96 × A_649_ − 0.32 × A_665_; Cx·c = (1000 × A − 2.05 × Ca − 114.8 × Cb)/245. Subsequently, the pigment content in the tissue (mg/g) was calculated using the following formula: Pigment content = Pigment concentration (calculated as above) × Extract volume × Dilution factor/FW (fresh weight) [48]. Proline content was determined using the Proline (Pro) Content Assay Kit (AKAM003C) provided by BoxBio (Beijing BoxBio Science & Technology Co., Ltd., Beijing, China). Malondialdehyde (MDA) content was determined using the Malondialdehyde (MDA) Content Assay Kit (AKFA013C) provided by BoxBio (Beijing BoxBio Science & Technology Co., Ltd.). The experimental procedures for these assays were performed according to the respective kit instructions. All experiments were conducted with three biological replicates to ensure reproducibility.

### 4.11. Statistic Analysis

The statistical analysis data were analyzed utilizing Microsoft Office Excel 2019 and IBM SPSS Statistics 26. To assess the variations among the samples, one-way analysis of variance (ANOVA) was conducted, followed by Tukey’s test (*p* < 0.01) for post hoc comparisons.

## 5. Conclusions

In general, we identified the CNGC gene family in eggplant and analyzed its expression patterns under cold stress. Finally, functional analysis was performed on the cold-responsive gene *SmCNGC1a*. These studies provide valuable information for the research on cold tolerance in eggplant. Further investigations can be based on these findings to explore the function of *SmCNGC* and the regulatory mechanisms of *SmCNGC1a* in cold tolerance in eggplant. This will provide a theoretical basis for the development of cold-tolerant eggplant varieties and breeding strategies aimed at enhancing cold tolerance in eggplant.

## Figures and Tables

**Table 1:** Identification and Physicochemical Analysis of *SmCNGC* Proteins.

Gene Name	Gene ID	Number of Amino Acid/aa	Molecular Weight	Theoretical pI	Instability Index	Aliphatic Index	Grand Average of Hydropathicity	Prediction of Subcellular Localization
SmCNGC1a	Smechr0500088.1	714	82,462.57	9.51	53.73	90.17	−0.208	cytomembrane
SmCNGC1b	Smechr0101357.1	708	81,926.22	9.32	48.72	93.4	−0.071	cytomembrane
SmCNGC1c	Smechr0302178.1	710	81,869.6	8.9	50.11	92.46	−0.132	cytomembrane
SmCNGC2	Smechr0202893.1	708	81,371.83	9.65	53.34	92.78	−0.034	cytomembrane
SmCNGC3	Smechr0400998.1	838	96,160.87	7.32	32.86	93.05	−0.198	cytomembrane
SmCNGC4a	Smechr1000491.1	685	80,107.48	8.28	46.96	90.31	−0.169	cytomembrane
SmCNGC4b	Smechr0400207.1	665	77,317.03	8.98	45.03	92.77	−0.075	cytomembrane
SmCNGC5	Smechr1200261.1	692	78,774.97	9.19	51.32	92.76	−0.114	cytomembrane
SmCNGC6	Smechr1100117.1	823	94,278.62	6.38	40.34	98.54	−0.131	cytomembrane
SmCNGC7	Smechr0700086.1	1074	122,281.25	9.3	53.27	90.8	−0.079	cytomembrane
SmCNGC8	Smechr0303133.1	689	79,669.91	8.78	43.35	91.13	−0.183	cytomembrane
SmCNGC9	Smechr0801568.1	482	55,399.66	8.21	37.08	96.24	−0.188	cytoplasm
SmCNGC10	Smechr0800622.1	714	82,222.56	5.82	38.97	95.03	−0.123	cytoplasm
SmCNGC11	Smechr0801458.1	859	96,677.4	6.6	40.65	98.43	−0.055	cytomembrane
SmCNGC12	Smechr0201396.1	655	75,003.7	7.84	39.83	93.45	−0.092	cytomembrane
SmCNGC13	Smechr0900702.1	612	69,109.84	7.32	46.02	99.1	0.033	cytomembrane
SmCNGC14	Smechr0801673.1	634	72,997.99	8.67	47.05	91.85	−0.097	cytomembrane
SmCNGC15a	Smechr0900248.1	659	76,020.09	8.73	49.81	93.38	−0.033	cytomembrane
SmCNGC15b	Smechr0600577.1	696	80,257.66	9.24	50.58	91.18	−0.19	cytomembrane
SmCNGC15c	Smechr1201893.1	704	80,655.83	9.26	53.81	88.82	−0.217	cytomembrane
SmCNGC16	Smechr0302884.1	674	77,929.8	8.31	47.48	89.39	−0.157	cytomembrane
SmCNGC17	Smechr0700014.1	720	83,011.82	9.32	41.97	93.74	−0.174	cytomembrane
SmCNGC18	Smechr0203033.1	689	79,438.12	7.17	49.98	88.78	−0.15	cytomembrane
SmCNGC19	Smechr0302451.1	840	95,490.13	6.12	39.05	97.96	−0.151	cytomembrane
SmCNGC20	Smechr0302478.1	771	88,894.76	9.32	49.87	89.29	−0.191	cytomembrane
SmCNGC21	Smechr0500154.1	827	94,588.85	6.62	39.78	94.79	−0.13	cytomembrane
SmCNGC22	Smechr0103753.1	629	72,010.19	8.16	44.82	99.63	−0.018	nucleus
SmCNGC23	Smechr1200076.1	884	99,814.77	6.47	39.65	96.24	−0.111	chloroplast
SmCNGC24	Smechr0100701.1	837	95,413.1	6.61	33.24	95.97	−0.13	cytomembrane

**Table 2:** Online Analysis Websites and URLs.

Website	URL
Eggplant Genome Database	http://eggplant-hq.cn/Eggplant/home/index (accessed on 3 March 2023)
InterPro protein family database	https://www.ebi.ac.uk/interpro/ (accessed on 3 March 2023)
NCBI CDD	https://www.ncbi.nlm.nih.gov/cdd/ (accessed on 3 March 2023)
Expasy ProtParam tool	https://web.expasy.org/protparam/ (accessed on 7 April 2023)
WoLF PSORT subcellular localization prediction tool	https://wolfpsort.hgc.jp/ (accessed on 7 April 2023)
TAIR	https://www.arabidopsis.org/ (accessed on 16 March 2023)
Tomato genome database	https://solgenomics.net/ (accessed on 16 March 2023)
Evolview v3	https://www.evolgenius.info/evolview/ (accessed on 16 March 2023)
MEME	http://memesuite.org/tools/meme/ (accessed on 30 March 2023)
MapGene2Chromosome v2.1	http://mg2c.iask.in/mg2c_v2.1/ (accessed on 30 March 2023)
PlantCARE	http://bioinformatics.psb.ugent.be/webtools/plantcare/html/ (accessed on 30 March 2023)

**Table 3:** Primers used in this study.

Gene Name	Gene ID	Forward Primer (5’ –> 3’)	Reverse Primer (5’ –> 3’)
SmCNGC1a	Smechr0500088.1	AACCAACGTTTAGCTCGTTGA	TAGAGGATGCATGCGAATTG
SmCNGC1b	Smechr0101357.1	AAAGCCACCAATCTGCTCAT	AGGAAAGGGATGCACATTGA
SmCNGC1c	Smechr0302178.1	CGGCAAATTTGGAGTGTTCT	TTTGGCCAGAAGGCAACTTA
SmCNGC2	Smechr0202893.1	CAACCTGATAACAGCGACGA	TCACAACTGGTGGAATGGAA
SmCNGC3	Smechr0400998.1	GGAAGTGAAATATTCATCATATGGTTT	CCACCTCTCTCACCGTACCT
SmCNGC4a	Smechr1000491.1	GGACAAGGATGTGGATGAGG	ACACGACCACGACCACTACA
SmCNGC4b	Smechr0400207.1	GCTCGAGTGATCTGATTGTTGA	TCCAAAATAAGTGATACCGATCC
SmCNGC5	Smechr1200261.1	TTGTTGATCTTTTGAGCTTTGC	TTTACACAATCGGTGTATATAAAACTC
SmCNGC6	Smechr1100117.1	TGGAGGTCGAGCAGAGTATG	TTTGCCGGCTAATTTTTCTC
SmCNGC7	Smechr0700086.1	GAGTCGAGTTTGAGGGCTTG	TCGCAGTCTTGCTGATGAAC
SmCNGC8	Smechr0303133.1	TTGGAGGGCAAAAAGAAAAG	TGGTTACATGCCCACCAGTA
SmCNGC9	Smechr0801568.1	CTGAAGGATCTGGATTCTTTGC	TCATCTTGACATCTTAACTTATGGA
SmCNGC10	Smechr0800622.1	GGACATGGAAAGCAAACCAA	CGTCCACAACTTTCACCTTC
SmCNGC11	Smechr0801458.1	ATTGCTTGTGGACAATGGTG	TCACCTCCATACCGGATGAT
SmCNGC12	Smechr0201396.1	GGGATTTGGAGGTTTTGGTT	TGTCCATCACCTTTCTCTTGC
SmCNGC13	Smechr0900702.1	TTTCAGAAATGTATCTGATTGACC	CTCAATGACTAGAATTCCGCTGT
SmCNGC14	Smechr0801673.1	AGCTGGCCAAAGAACTTTACA	TGTTGATCATCCTCGGGTTC
SmCNGC15a	Smechr0900248.1	CCTCGAGGAGGTCCTATAAACA	CCATGGGATAACTTGCATCC
SmCNGC15b	Smechr0600577.1	CAGTTGTAACTTGTAAGATAAGATGGA	ATGGCACAAAAGCTGCAGTA
SmCNGC15c	Smechr1201893.1	CAAATGTGGAAGGGTGTTTT	GTTTCTCTTCCCCCTCTTGC
SmCNGC16	Smechr0302884.1	CAGGGAAAGTCGTTTTGGAA	GGAAGCAGCAAAAACAGAGG
SmCNGC17	Smechr0700014.1	TGGGAGGAAAAGCAGACAGT	CCTTTTTAGGCCTCCCAAAC
SmCNGC18	Smechr0203033.1	GGTGGCGTCAGATTTTTGAT	TGACGAAAGGGACGAAGAAG
SmCNGC19	Smechr0302451.1	GCCAAAGAAGTTCAGGCAGA	AGTAATTCCGCAGCCATTTG
SmCNGC20	Smechr0302478.1	TTGGTCGAGAGCCTGAGAAT	TACGCCAACCATTTCGTTCT
SmCNGC21	Smechr0500154.1	AATCGTCGAGAAGCAGCAGT	GAGGCCATTGATGACGTTTT
SmCNGC22	Smechr0103753.1	AAAAACAGAGGAAACAAATATAATGAA	TGCTATCATGTTCATCTCATTACCA
SmCNGC23	Smechr1200076.1	TGGAGCAGCACAAGAAATTG	TTGCCGATCATAAGGTGAAA
SmCNGC24	Smechr0100701.1	TGCAAATGAGCCATTCATACA	TGCTACTCCCATGGCTATCA

## Data Availability

Not applicable.
